# ECORISK2050: An Innovative Training Network for predicting the effects of global change on the emission, fate, effects, and risks of chemicals in aquatic ecosystems

**DOI:** 10.12688/openreseurope.14283.1

**Published:** 2021-12-20

**Authors:** Samuel A. Welch, Taylor Lane, Alizée O.S Desrousseaux, Joanke van Dijk, Annika Mangold-Döring, Rudrani Gajraj, John D. Hader, Markus Hermann, Anju Parvathi Ayillyath Kutteyeri, Sophie Mentzel, Poornima Nagesh, Francesco Polazzo, Sabrina K. Roth, Alistair B.A. Boxall, Benny Chefetz, Stefan C. Dekker, Josef Eitzinger, Merete Grung, Matthew MacLeod, S. Jannicke Moe, Andreu Rico, Anna Sobek, Annemarie P. van Wezel, Paul van den Brink

**Affiliations:** 1 Norwegian Institute for Water Research, Oslo, 0579, Norway; 2 Environment Department, University of York, Heslington, York, UK; 3 Copernicus Institute of Sustainable Development, Utrecht University, Utrecht, The Netherlands; 4 Aquatic Ecology and Water Quality Management Group, Wageningen University, Wageningen, 6700 AA, The Netherlands; 5 Institute of Meteorology and Climatology, Department of Water, Atmosphere and Environment (WAU), University of Natural Resources and Life sciences (BOKU), Vienna, Austria; 6 Department of Environmental Science, Stockholm University, Stockholm, 106 91, Sweden; 7 Department of Soil and Water Sciences, The Hebrew University of Jerusalem, Rehovot, 7610001, Israel; 8 IMDEA Water Institute, Science and Technology Campus of the University of Alcalá, Alcalá de Henares, Madrid, 28805, Spain

**Keywords:** climate change, ecotoxicology, innovative training network, emerging chemicals, agricultural pollution, PCPPs, global change

## Abstract

By 2050, the global population is predicted to reach nine billion, with almost three quarters living in cities. The road to 2050 will be marked by changes in land use, climate, and the management of water and food across the world. These global changes (GCs) will likely affect the emissions, transport, and fate of chemicals, and thus the exposure of the natural environment to chemicals.

ECORISK2050 is a Marie Skłodowska-Curie Innovative Training Network that brings together an interdisciplinary consortium of academic, industry and governmental partners to deliver a new generation of scientists, with the skills required to study and manage the effects of GCs on chemical risks to the aquatic environment. The research and training goals are to: (1) assess how inputs and behaviour of chemicals from agriculture and urban environments are affected by different environmental conditions, and how different GC scenarios will drive changes in chemical risks to human and ecosystem health; (2) identify short-to-medium term adaptation and mitigation strategies, to abate unacceptable increases to risks, and (3) develop tools for use by industry and policymakers for the assessment and management of the impacts of GC-related drivers on chemical risks.

This project will deliver the next generation of scientists, consultants, and industry and governmental decision-makers who have the knowledge and skillsets required to address the changing pressures associated with chemicals emitted by agricultural and urban activities, on aquatic systems on the path to 2050 and beyond.

## Introduction

### Disclaimer

The views expressed in this article are those of the author(s). Publication in Open Research Europe does not imply endorsement of the European Commission.

***Background and aims of ECORISK2050*.** As the human population develops and evolves throughout the 21 ^st^ century, certain global changes (GCs) are expected to occur, that could modify the way humans and the environment are exposed to chemicals ( EEA, 2015). Intensifying urbanisation is of increasing concern, as growing cities are areas of high population density where chemical consumption is rising, concentrated, diverse, and difficult to manage ( Brooks, 2019). Furthermore, the world’s growing and urbanising population will require greater clean water supplies and larger quantities of crop and livestock foodstuffs to be produced through agriculture. These challenges will in turn drive the development and usage of novel irrigation methods, fertilizers, pesticides, and pharmaceuticals to adapt to new pests, diseases, and climatic factors ( FAO, 2018; van Vuuren *et al.*, 2019; Webb & Buratini, 2016). Thus, technology such as the use of second and third generation nanomaterials, more efficient water reuse techniques, or a transition towards using novel treatments for disease such as biologics, are likely to be increasingly relevant in the near future ( Camboni *et al.*, 2019).

Climate change (CC), in unison with other GCs, will affect the overall emissions, environmental transport pathways, and fate of chemicals, changing how chemical exposure occurs in the environment and humans ( Bloomfield *et al.*, 2006; Boxall *et al.*, 2009; Gouin *et al.*, 2013). Climate change is anticipated to provoke a range of effects across various levels of biological systems, causing changes at the individual level, such as organisms having an increased or decreased sensitivity to chemicals, shifts at the population level due to altered feeding strategies, and a restructuring of ecosystems due to changes in trophic level complexity and resilience ( Hooper *et al.*, 2013; Moe *et al.*, 2013). Certain aspects of CC are of specific interest, such as the effects of overall increased temperatures, which has been previously demonstrated to enhance the toxicity of certain, but not all, chemical stressors ( Arenas‐Sánchez *et al.*, 2019; Cairns *et al.*, 1978). In addition, extreme weather events, which includes heatwaves, droughts, intense precipitation, and floods, are forecast to occur more frequently and with greater severity in the future, and these events have the disposition to alter the mobility and ecological interactions of chemicals in and between agricultural and aquatic environments, providing potential pathways for chemical exposure in previously uncontaminated areas ( Jiménez Cisneros *et al.*, 2014).

Confronting the challenge of evaluating how GC will affect chemical exposure and risks of wildlife, humans, and the environment they inhabit, requires tackling uncertainty and addressing open questions ( Brack *et al.,* 2015; Chapman, 2018; Holmstrup *et al.,* 2010). For instance, the types and concentrations of chemicals released into the environment, the locations and to degrees of exposure to these chemicals, and the vulnerability of communities to climate and chemical stressors in different regions of the European Union could be different under future environmental conditions compared to today ( Balbus *et al.,* 2013; Moe *et al.,* 2013). Modern testing approaches and models used for risk assessment of chemicals are not designed for predicting future risks, as they do not take a systems approach, and thus often disregard temperature-fate and effect relationships, novel transport pathways such as (waste)water-reuse scenarios, and the effects of climate-induced stressors on biodiversity and ecosystem functions ( Carter *et al.,* 2019; Hooper *et al.,* 2013; Su *et al.,* 2019). Lastly, current approaches to environmental risk assessment (ERA) tend to compartmentalize risks, primarily considering the risks of chemicals in different applications in isolation, and ecological risks are typically treated separately from human health risks ( Ågerstrand & Beronius, 2016).

The ECORISK2050 innovative training network was initiated with support from the European Union Horizon 2020 program to support the European Union goal of a non-toxic environment by 2050. Taking a holistic approach to address the many challenges in chemical risk assessment under GC, ECORISK2050 brings together an interdisciplinary team of experts in global change, scenario development, environmental modelling, environmental chemistry, exposure assessment, ecology, ecotoxicology, and risk assessment, drawing on intersectoral input from academic beneficiaries and key stakeholders from the chemical and water industries and regulatory sectors. This paper provides an overview of the ECORISK2050 projects as well as the individual sub-projects and scientific questions that will be addressed. The specific research objectives of the project are:

- To assess how the inputs of chemicals from agriculture and urban environments and their fate and transport will be affected by GC for different European scenarios, in order to assess changes in risks to human and ecosystem health.
- To identify potential adaptation and mitigation strategies, which can be implemented in the short and medium term, to abate unacceptable changes in risks, and use the GC scenarios to develop robust implementation pathways for these strategies.
- To develop a set of tools for use by industry and policymakers that allow the impacts of a range of GC-related drivers on chemical risks to be assessed and managed.

**Figure 1.**
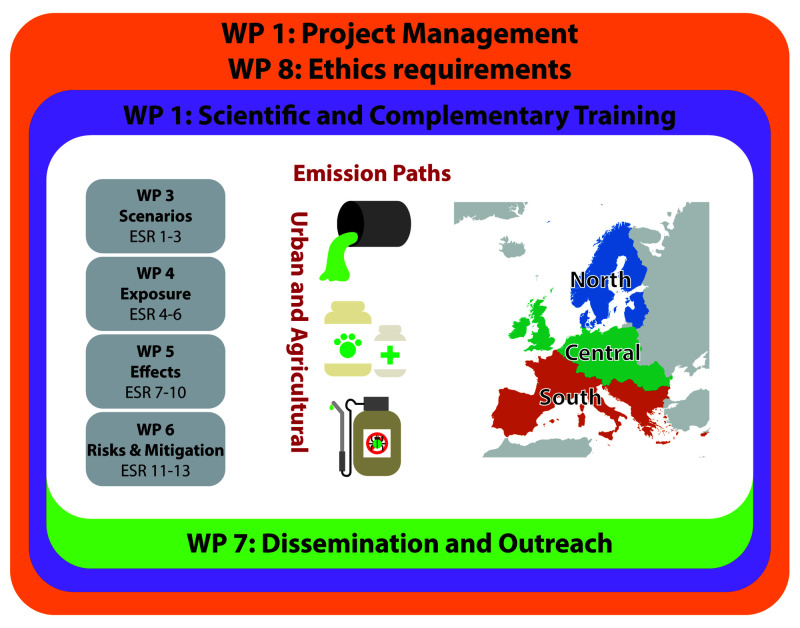
Schematic overview showing the Work Packages and ESR projects included in ECORISK2050.

***Project structure*.** ECORISK2050 has four interrelated research work packages (WPs 3-6; Figure 1; Table 1) and three additional WPs (WPs 1, 2, and 7) which focus on management, training, and ensuring impact. The research WPs will address the interaction between global change and environmental risks by developing scenarios for the future and assessing changes in exposure and effects, which will then be integrated in a risk and mitigation WP ( Figure 1). Scenarios will include both agricultural and urban environments located in the southern, central, and northern regions of Europe ( Figure 1).

**Table 1.** Description of the objectives and expected outputs of the four experimental and modelling work packages in the ECORISK2050 project.

Work package	Objectives	Expected output
Scenarios (WP 3)	• Develop chemical emission scenarios of agricultural and urban systems in Europe under global change, considering circular economy and non-toxic environment options • Develop agronomic land use and management scenarios for different representative climate and socioeconomic scenarios • Identify main pest pressures considering farm management options under the named scenarios • Estimate chemical inputs under different environmental characteristics of selected European agricultural and urban systems	• Global climate/land use/agronomic management and emission scenarios downscaled to the three case study regions • Chemical emission scenarios of urban systems in selected case study regions • Chemical emission scenarios from pest management options under scenarios in selected agricultural systems
Exposure (WP 4)	• Develop models for estimating the effects of environmental change on the fate of chemicals in aquatic environments and uptake into organisms with different species traits • Develop new models for estimating changes in human exposure to chemicals via crop items resulting from an increase in wastewater re-use systems • Develop an exposure modelling framework for estimating concentrations of emerging contaminants in river basins, drinking water, and fish/shellfish, now and in the future	• Modelling approaches for estimating the effects of key environmental change parameters on the fate and uptake of chemicals • Crop uptake models and human exposure models for contaminants in water re-use systems • Exposure modelling framework for estimating effects of environmental change on chemical exposure in river basins and drinking water • Future ecological and human exposure scenarios for chemical contaminants
Effects (WP 5)	• Assess and compare the sensitivity and vulnerability of aquatic populations and communities to global change and chemical pollution under different geographical and biological (community composition) scenarios • Quantify the impacts of chemicals on key ecological functions • Develop a set of validated *in silico* tools to evaluate the effects of the chemicals at different levels of biological organisation	• Comparison of sensitivity and vulnerability to climate change and chemical stress in different geographical regions • New framework for evaluating the influence of community structure and global change on the vulnerability to chemicals • EU-level chemical vulnerability maps and scenarios • New framework for assessing the resilience of microbial communities to chemical stress • Determination of the chemical effects on microbial ecological functions and chemical tolerance limits • New modelling framework for assessing the combined effects of chemical and global change stressors
Risk and Mitigation (WP 6)	• Assess and compare the sensitivity and vulnerability of aquatic populations and communities to global change and chemical pollution under different geographical and biological (community composition) scenarios • Quantify the impacts of chemicals on key ecological functions • Develop a set of validated in silico tools to evaluate the effects of the chemicals at different levels of biological organisation	• Comparison of sensitivity and vulnerability to climate change and chemical stress in different geographical regions • New framework for evaluating the influence of community structure and global change on the vulnerability to chemicals • EU-level chemical vulnerability maps and scenarios • New framework for assessing the resilience of microbial communities to chemical stress • Determination of the chemical effects on microbial ecological functions and chemical tolerance limits • New modelling framework for assessing the combined effects of chemical and global change stressors

**Figure 2.**
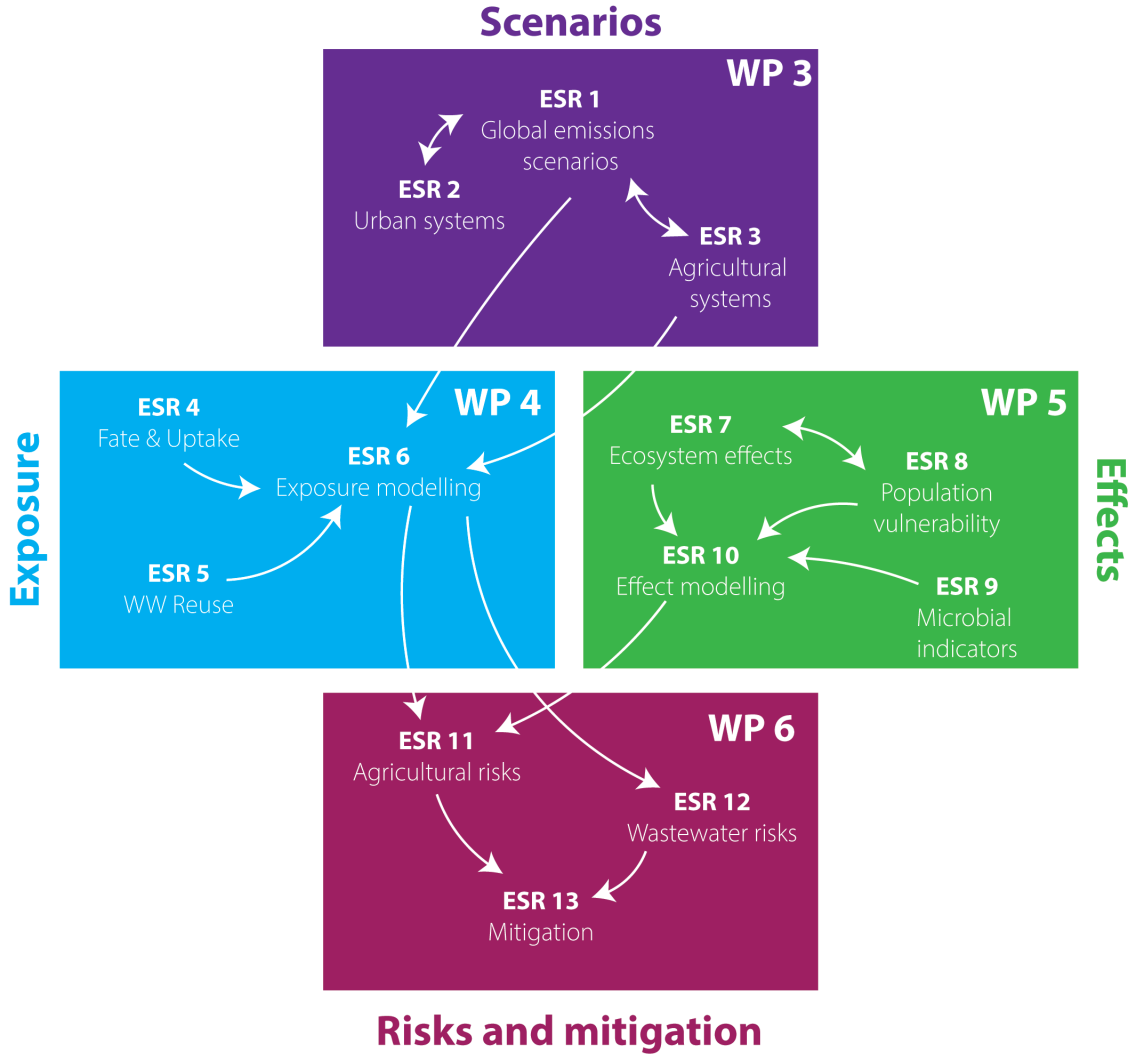
Scheme illustrating the interlinkages between the 13 ECORISK2050 ESR projects.

The ECORISK2050 project comprises 13 sub-projects each delivered by an early-stage researcher (ESR), distributed across four research WPs ( Figure 2). These sub-projects cover scenario development, modelling studies, laboratory and semi-field-based fate and effect studies, as well as environmental risk assessment and management approaches, to understand how chemical risks will change in a range of GC scenarios, and what effective solutions can be implemented.

The 13 ESRs are hosted and supervised by eight beneficiaries (see affiliations of authors) and co-supervised by 16 partner organisations. The beneficiaries include six universities and two research institutes, located around Europe (The Netherlands, United Kingdom (UK), Norway, Sweden, Austria, Spain) and in Israel. The partner organisations include research institutes, companies, governmental agencies, a consultancy firm, a university, and a wastewater treatment organisation. The early-stage researchers are all enrolled in a PhD program.

***Work package 3: scenarios*.** Scenarios – projections of different possible futures ( Rosentrater, 2010) – have been used by the scientific community for decades to analyse climate change ( van Vuuren *et al.*, 2012). They are an essential tool for stakeholders – politicians, public institutes, and private companies – to aid effective decision-making and move towards a desired future ( Pilli-Sihvola *et al.*, 2015). The scenario work package will develop qualitative and quantitative scenarios for chemical emissions at urban, agricultural, and regional scales in the future. Chemical emissions will be studied and divided into five categories with different drivers and sources: pharmaceuticals, personal care products, pesticides, industrial chemicals, and food chemicals. ECORISK2050 scenarios will be based on shared socio-economic pathways (SSPs), a scenario framework for socio-economic change research. SSPs are qualitative scenarios describing five alternative future societies based on changes in demographics, human development, economics and lifestyles, technology, and environment and natural resources. These drivers lead to societies where adaptation to challenge and mitigation of impacts range from easier to harder to implement. Representative concentration pathways (RCPs) - quantitative projections of future levels of greenhouse gases - will also be used, and the combination of SSPs and RCPs will allow us to study possible future societies in the context of projected greenhouse gas concentrations ( O’Neill *et al.*, 2014). Within ECORISK2050, scenarios will be specifically adapted to urban, agricultural, and regional scales, and to chemical emissions, which will address difficulties stakeholders have in using scenarios that are not relevant or specific enough to individual contexts ( Carlsson *et al.*, 2018). This will require that drivers to be adapted to chemical emissions and to specific types of chemicals. Thus, we aim to study and quantify chemical emissions scenarios, under not only climate change, but socio-economic, technological, and lifestyle change as well. To summarise, pharmaceuticals, personal care products, pesticides, industrial chemicals, and food chemical scenarios will be developed based on SSPs and RCPs. These scenarios will focus on developing qualitative and quantitative measures of global change at the following spatial scales:

•    Global chemical emission scenarios downscaled for three European regions located in northern, central, and southern Europe, specifically driven by agronomic land-use changes and management.

•    Local-level scenarios focusing on chemical emissions in urban environments.

Chemical emission scenarios will then be used to drive models for the exposure, effect, and risk assessment packages.

**ESR 1: Global chemical emission scenarios**

The high production and widespread use of chemicals for a range of applications has resulted in their continuous release and omnipresent distribution in the environment ( Bai *et al.,* 2018). A key challenge in assessing the risk of chemical pollution and their environmental impact is producing reliable estimates of chemical releases into the environment ( Arnot *et al.,* 2012; Douziech *et al.,* 2018). The sheer number of chemicals and range of emissions sources (industrial, agricultural, residential, among others) makes it a complex task to estimate chemical emissions. At present, there are over 350,000 chemicals and mixtures of chemicals registered for production and use ( Wang *et al.,* 2020), with continuous addition of new chemicals in the market ( van Wezel *et al.,* 2017).

The use of chemicals is expected to grow rapidly driven by drivers such as demographic change ( Bunke *et al.,* 2019), population growth, change in consumption patterns and climate change ( Mateo-Sagasta *et al.,* 2018), which could lead to further emissions of chemicals, posing significant water quality concerns. This creates the need to understand the multitude of chemical emissions over time. To better understand how the future chemical emissions will change, it is important to understand how socio-economic drivers will influence the production and use of chemicals. Socio-economic scenario analysis has been a useful tool in investigating the long-term consequences of anthropogenic change and mitigation options ( Kriegler *et al.,* 2012).

**Figure 3.**
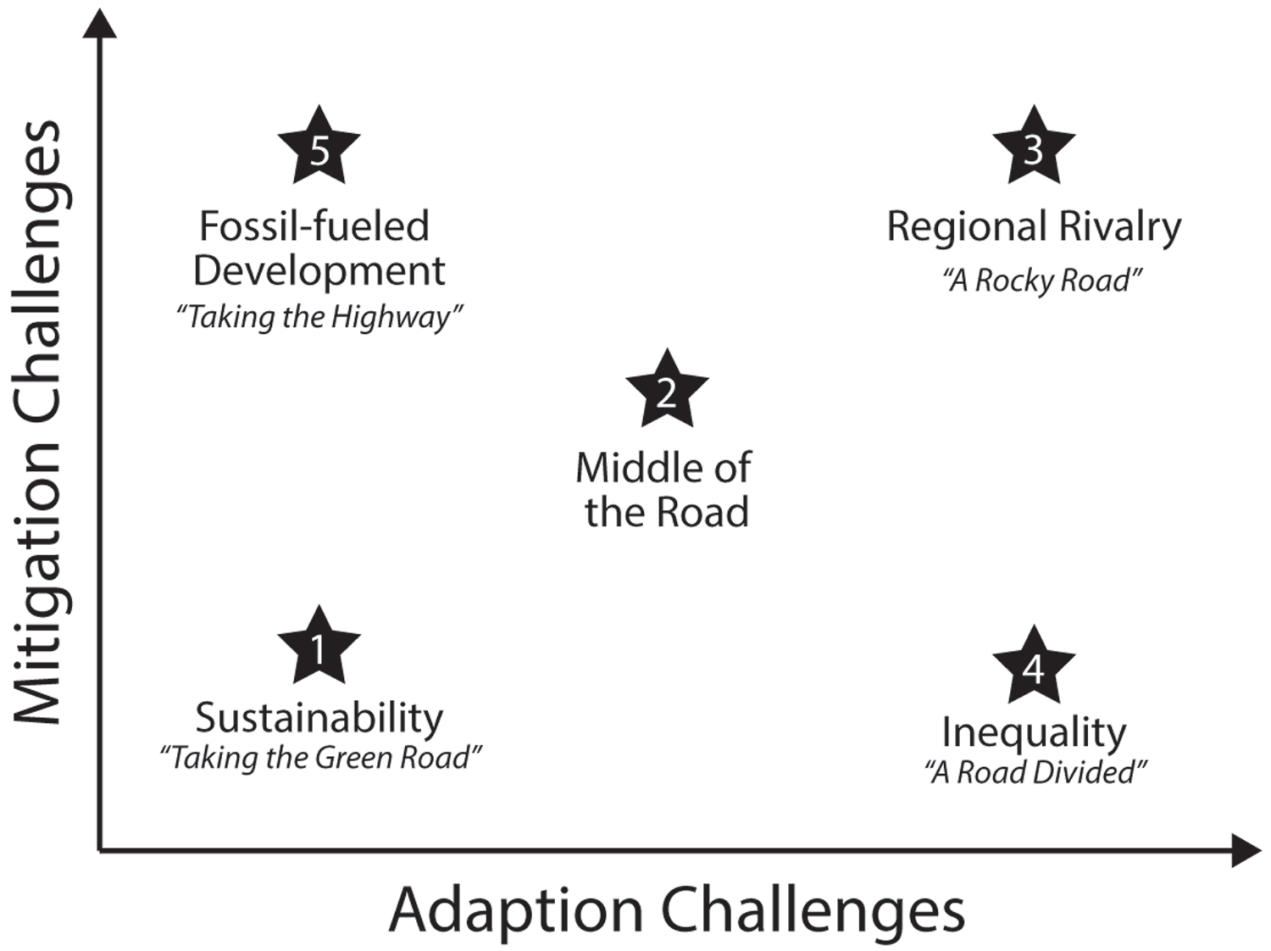
Five SSPs representing different combinations of challenges to mitigation and to adaptation. Original work, based on O’Neill *et al.*, 2014.

In this component of the ECORISK2050 project, socio-economic scenario analysis will be used to understand the past, current, and possible chemical emission trends that are mediated by socio-economic drivers. SSPs from the Integrated Model to Assess the Global Environment (IMAGE3.0) model will be used as input for our empirical emission models to develop emission scenarios for chemicals ( Figure 3). Regional and global emission scenarios will include three SSP scenarios: SSP1 ("Sustainability"), SSP2 ("Middle of the Road") and SSP3 ("Regional Rivalry"), including changes in animal husbandry. Future chemical emissions will be estimated based on emission factors and activities (e.g., total population, GDP, harvested area, crop production, feed stock, sector value added, among others) for industrial chemicals, pesticides, and pharmaceuticals in Europe. Regional emission scenarios will focus on three different regions (northern, central, and southern Europe) for to the future until the year 2100. The developed chemical emission scenarios will be used to model the combined chemical load of multiple chemicals on surface waters and their impacts on water quality. Furthermore, target scenarios will be developed describing mitigation efforts

**ESR 2: Urban emission scenarios**

Cities and other urban environments are attractive areas to live with access to public services, employment, culture, and other types of leisure ( Ruth & Franklin, 2014). The high concentration of population and industries in urban areas requires the use of great quantities of chemicals, causing chemical pollution in the surrounding aquatic environment. This pollution usually results from ineffective wastewater treatment, or direct release into the environment through leakage ( Petrie *et al.,* 2015). From heavy metal-contaminated cigarette butt leachate causing cellular abnormalities in mussels ( Montalvão *et al.,* 2019), to air pollution provoking the development of respiratory and cardiovascular diseases in humans ( Sciaraffa *et al.,* 2017), urban chemical pollution is causing worldwide adverse effects and is negatively affecting cities’ liveability ( Martínez-Bravo *et al.,* 2019).

Present and future risks of chemical pollution in cities will be studied in ECORISK2050 with spatial and temporal models and scenarios development. Chemical emissions for the year 2050 will be studied for the cities of York (UK), Madrid (Spain), and Oslo (Norway). To focus scenarios and models on the most toxic chemicals, prioritisation of chemicals in urban environments will first be conducted based on published measurements of pollutant concentrations in cities, and a priority list of chemicals for urban environments will be developed. A one-year monitoring of rivers in York, Madrid and Oslo will then be conducted upstream, within, and downstream of the city centre and wastewater treatment plants. A spatial and temporal model to estimate current chemical emissions to the aquatic environment will then be constructed using data collected.

To analyse different emission situations up to 2050, local urban scenarios will be developed. First, SSPs will be downscaled to local scenarios. At local workshops, stakeholders, such as public representatives, local business leaders, retailers, schoolteachers, and wastewater treatment engineers, will provide input to the development of qualitative scenarios that will represent realistic storylines for development up to 2050. While the three cities’ scenarios will be driven by common large-scale factors, demographic and geographic specificities of each city will also be implemented. These scenario storylines for each city will then be translated into corresponding emission scenarios and chemical concentration trends in the environment by 2050. The list of priority chemicals, scenarios and modelled concentrations will be shared with exposure, effect, and risk assessment work packages as a basis for assessment on the species and community level.

**ESR 3: Agricultural emission scenarios**

Agricultural chemicals include chemical fertilisers and plant protection products (PPPs) such as herbicides, insecticides, and fungicides, and are used to stimulate crop productivity ( Lamichhane *et al.,* 2016). Their residues pose a significant threat to small streams and catchments ( Beketov & Liess, 2008). Pesticide consumption in the EU continues to increase in spite of rising restrictions on their use ( Jess *et al.,* 2014; Lamichhane *et al.,* 2016), due to the increased metabolic activity, reproduction, and survival of pests ( FAO, 2019). Simultaneously, climate change is expected to further affect crop yields, soil fertility, pesticide use, and nutrient runoff in Europe ( Deutsch *et al.,* 2018; Eitzinger *et al.,* 2013; Kocmánková *et al.,* 2009; Olesen *et al.,* 2011).

Predicted impacts of specific pests on agricultural production under emission scenarios determined by simulation models covering shorter time periods have been made available ( Eitzinger *et al.,* 2013; FAO, 2019) but are still under evaluation ( Deutsch *et al.,* 2018; Svobodová *et al.,* 2014). Entomologists face several challenges in modelling pest impacts, and have proposed semi-quantitative and quantitative approaches to determine the abundance, distribution, phenology, physiology, population dynamics, seasonal dynamics, and timely forecasts (short-term, and long-term) of pests. Existing insect modelling approaches usually focus on individual components of the system devoid of crop growth requirement and management patterns ( Tonnang *et al.,* 2017). Quantitative pest simulation models are required to build predictions on pest risks under different climate change and crop management scenarios. However, many current approaches do not consider meteorological drivers, and are not connected to chemical emission rates, or show little accuracy in simulating emission rates at high temporal resolutions. For the ECORISK2050 project, the database of selected agroecosystems from case study regions (Norway, Austria) for pest models will be established, followed by identification and investigation of critical chemical emission risks and pest pressures, under the applied scenario combinations of climate and crop management.

A modelling framework is required which uses daily gridded meteorological data and spatially varying soil and agricultural practice data for simulating processes in the soil, atmosphere, and vegetation compartments ( Li & Jin, 2013). SSP scenarios will be used to estimate future emission factors and for downscaling to the case study regions in Norway and Austria ( Li *et al.,* 2005). At the regional scale, scenarios for site-specific cropping patterns, pest risks and multiple weather-related risk assessment will be examined. Agricultural emission scenarios will be modelled, including a reference scenario based on SSPs, climate change scenarios based on the outputs of general circulation models (GCMs) using RCP 2.6, 4.5, and 8.5, and existing crop and pest models.

***Work package 4: Exposure*.** Modelling the chemical fate of environmental pollutants, from source to receptor, offers risk assessors and regulators the predictive capabilities required to make knowledge-based decisions that are protective of ecological and human health ( Di Guardo *et al.,* 2018; Su *et al.,* 2019). Various direct and indirect factors related to GC over the coming decades are expected to influence the emissions, fate, and transport of environmental pollutants, not only through alterations to the environment but also through shifts in demographic and socio-economic patterns, and this will alter chemical exposure routes for humans and the environment ( Balbus *et al.,* 2013).

For instance, a review by Lamon *et al.* (2009) discussed possible ways that changes in temperature, precipitation, and other environmental factors could influence the behaviour and fate of persistent organic pollutants in the environment. While several modelling studies have investigated the impact that changes in some of these environmental factors could have on chemical fate and transport, and found modest impacts (changes up to an order of two to three-fold), these studies focused on neutral organic compounds rather than ionizing compounds ( Gouin *et al.,* 2013; Kong *et al.,* 2014). Furthermore, extreme weather events are becoming more frequent as a consequence of CC and the associated implications for chemical fate and transport, particularly from intense precipitation events, warrants further development of modelling scenarios ( Boxall *et al.*, 2009). Additionally, the use of reclaimed wastewater and biosolids in agriculture is forecast to increase in the future, thereby altering the emissions of contaminants to the environment and creating a need to investigate how the type, fate, and transport of chemicals from these emission sources may change under projected climate conditions ( Boxall *et al.*, 2009; Carter *et al.*, 2019; Fu *et al.*, 2019).

Ultimately, existing exposure modelling approaches have so far not fully captured all anticipated aspects of GC ( Boxall *et al.,* 2009; Di Guardo *et al.,* 2018). Therefore, specific research aims of ECORISK2050 are to:

•    Develop models for estimating the effects of environmental change on the fate, uptake, and metabolism of pharmaceutical and agricultural chemicals in aquatic systems and organisms under future water chemistry parameters.

•    Develop new models for estimating changes in human exposure to pharmaceuticals and agricultural chemicals via crop items resulting from an increase in wastewater reuse systems; and

•    Develop a fate, transport, and exposure modelling framework for estimating concentrations of pharmaceuticals and agricultural chemicals in river basins, drinking water, and fish/shellfish, as well as the present and future associated human and ecological exposure concentrations.

By combining the parameterisation work with the modelling frameworks and emissions scenarios (WP 3), the research discussed in this section will provide the exposure information required for the risk assessment and mitigation work (WP 6) performed by ESRs 11-13.

**ESR 4: Fate, uptake, and metabolism of novel contaminants in aquatic environments**

Freshwater environmental quality leading up to 2050 and beyond is anticipated to shift in response to aspects of GC such as land use conversions related to urbanisation, agricultural practices, environmental pollution, and climate change ( Gouin *et al.,* 2013; Jiménez Cisneros *et al.,* 2014). For example, increases in surface water temperatures, increased loads of organic carbon, decreases in freshwater pH, and increases in salinity for systems that are prone to saltwater intrusion, are all possible or likely in the future ( Hasler *et al.,* 2018; Hoegh-Guldberg *et al.,* 2018; Webb & Nobilis, 2007; Noacco *et al.,* 2017).

Chemical fate processes in aquatic environments such as sorption, volatilisation, degradation, and bioaccumulation, can be influenced by changes in these factors; as an example, temperature increases the rate of chemical and biological reactions, while pH affects the uptake of ionisable chemicals ( Gouin *et al.,* 2013). Incorporating the various effects that changes in these environmental conditions can have on chemical fate processes into multimedia fate and exposure models is becoming increasingly relevant ( Di Guardo *et al.,* 2018), particularly for an expanded chemical domain which focuses on ionisable chemicals and chemical transformation products ( Franco *et al.,* 2010). 

Therefore, the ECORISK2050 project aims to determine how predicted shifts in future environmental conditions will influence the fate of emerging chemical contaminants representing a range of physicochemical properties. Bioaccumulation and the metabolism of chemical compounds will also be investigated in freshwater organisms with contrasting species-specific traits. Conjointly, the fate and uptake studies will support generating modelled predictions for external and internal exposure concentrations of emerging chemical contaminants of concern under future environmental conditions. Experimental studies will also evaluate the effects of changing environmental conditions (e.g., temperature and moisture content) on key fate processes such as sorption and biodegradation.

**ESR 5: Agricultural systems and wastewater reuse exposure**

Water shortage is one of the most serious environmental issues faced worldwide ( Sapkota, 2019) and is an especially major concern in arid and semi-arid regions. Water-related issues are increasing with GCs like population growth, altered weather patterns and increased pollution. Meanwhile, agricultural sectors contribute 92% of global water consumption ( Hoekstra & Mekonnen, 2012). Thus, wastewater irrigation has been identified as an efficient practice to manage the enormous water stress in agricultural practices ( Goldstein *et al.,* 2014). In Israel, more than 70% of the wastewater produced is reused, and more than 85% of treated wastewater is used for agricultural applications ( Bixio *et al.,* 2006; Goldstein *et al.,* 2014).

Simultaneously, pharmaceutical and personal care products (PPCPs) have become an integral part of day-to-day life. Consequently, the parent compounds and by-products of PPCPs have been detected in domestic wastewater ( Ternes *et al.,* 2007). Conventional treatment technologies are not sufficient to completely remove PPCPs from wastewater ( Andreozzi *et al.,* 2004), meaning these chemicals can leach into the soil through wastewater irrigation. PPCPs and their metabolites, having low sorption affinity and high mobility in soil, can contaminate nearby sources of water ( Paz *et al.,* 2016). Those pharmaceutical compounds that have a high resistance to biodegradation will accumulate in soil, and as a result, their uptake by plants will increase ( Grossberger *et al.,* 2014). Furthermore, pharmaceutical compounds will accumulate in higher concentrations in crops grown in soils with low organic matter and clay content ( Ben Mordechay *et al.,* 2018; Goldstein *et al.,* 2014). The presence of PPCPs and their metabolites in the agricultural environment raises concerns due to the potential ecological and health risk associated with human exposure to these pollutants ( Paltiel *et al.,* 2016; Schapira *et al.,* 2020). These risks can be more serious in countries where raw wastewater or semi-treated wastewater is used for irrigation ( Carter *et al.,* 2019).

Knowledge about the uptake, accumulation, and transformation potentials of PPCPs in plants, as well as plant uptake of PPCPs in various soil conditions, is limited ( Fu *et al.,* 2019; Wu *et al.,* 2015). An extensive study on soil, water, and food contamination by PPCPs would be beneficial for understanding the potential human health risk and degree of treatment required for the agricultural reuse of wastewater. This experimental work within the ECORISK2050 project will explore the fate and behaviour of pharmaceutical compounds in the agricultural environment, and these data will be used to develop crop uptake and exposure models for wastewater reuse farming systems.

**ESR 6: Human and aquatic environment exposure scenarios**

Linking of the GC scenarios developed within ECORISK2050 to the fate, transport, and uptake of pharmaceuticals and agricultural chemicals in aquatic environments will be accomplished using exposure modelling framework at a range of spatial scales. Modelling scenarios will consider aquatic and land-based fate processes, direct aquatic emissions from STPs, and emissions due to the application of pesticides, reused wastewater, and biosolids to agricultural fields. Local/catchment-scale modelling will likely employ a multimedia watershed chemical fate model such as the INCA-Contaminants model ( Nizzetto *et al.,* 2016). Such models are capable of describing transient/extreme precipitation events on chemical fate; this environmental factor is likely to change due to CC (e.g., Boxall *et al.*, 2009) but its impacts on the chemical fate of a wide variety of chemicals is not well established. Regional/continental-scale fate and transport modelling will consider emissions from European sewage treatment plants (as in Grill *et al.* (2016)) as well as runoff emissions from agricultural regions and will likely utilise a version of the BETR modelling framework ( Gouin *et al.,* 2013; Wöhrnschimmel *et al.,* 2013) with possible enhancements, to better represent European river and lake environments. Emissions from STPs can be determined using the SimpleTreat 4.0 model ( Struijs, 2014), accounting for changes to the influent wastewater resulting from global change (e.g., temperature and population increase). Environmental parameters used in the chemical fate modelling will reflect various CC scenarios (e.g., Kong *et al.*, 2014) and the impacts of changes in long-term averages as well as changes in short-duration events on chemical fate will be explored. Fate and exposure modelling will be conducted in the context of a chemical property space, for the range of properties associated with pharmaceuticals and agricultural chemicals (similar to Gouin *et al.*, 2013; and Wöhrnschimmel *et al.*, 2013); however, integrated exposures can also be determined considering the projected use of chemicals with specific properties.

Using the ACC-HUMAN model ( Czub & McLachlan, 2004) or a similar bioaccumulation model along with country-resolved ingestion rates of fish and shellfish (e.g., EFSA, 2011), ingestion exposure estimates will be derived for humans. Estimated removal of chemicals through surface water purification could also be considered to estimate drinking water exposures. The exposure estimates derived from this modelling, combined with the agricultural-based ingestion exposures estimated by ESR 4, will provide a complete picture of human exposure to ingestion of pharmaceuticals and agricultural chemicals under the GC scenarios.

***Work Package 5: Effects*.** The influence of anthropogenic activities and climate change is not only expected to modify chemical exposure, but also to be responsible for dramatic changes to the physiology of aquatic organisms and the structure of aquatic ecosystems, which can influence their sensitivity and recovery capacity to chemical stress ( Moe *et al.,* 2013). For example, an increase in water temperature is generally related to a higher toxicity of chemicals, but also contributes to shorter recovery times in affected populations ( Arenas‐Sánchez *et al.,* 2019). On the other hand, future alterations of water temperatures, nutrient availability and hydrological regimes are expected to affect species interactions and to modify the structure of aquatic communities ( Jackson *et al.,* 2016), thus influencing their biological trait distribution and their resilience to specific chemical stressors.

The extent to which GC stressors are going to affect ecosystems in Europe is not spatially homogeneous, and the tolerance of aquatic organisms to the combination of GC stressors and chemical pollutants will likely also vary across biogeographic regions ( Van den Berg *et al.,* 2019). Thus, any investigation of the responses of aquatic ecosystems to chemical pollution requires the evaluation of aquatic organisms under local exposure and stress conditions. For this purpose, novel approaches, and tools to manipulate environmental variables in multiple species test settings (micro and mesocosms) will be developed within ECORISK2050. Furthermore, the extent to which aquatic communities can tolerate the influence of complex chemical mixtures and their capacity to maintain ecosystem functions will be investigated to assess the potential for disruption of ecosystem services in alternative future scenarios.

The aim of this WP will be to investigate the interactive effects of GC stressors and chemical pollution on ecosystem structure and function, and to develop a set of tools that allow the integration of GC on future chemical effect assessments and on the derivation of environmental quality standards. Particularly, the work programmed within this WP is intended to:

•    Describe the interactive effects of GC stressors and chemical pollution on aquatic populations and communities.

•    Compare the sensitivity and vulnerability of aquatic ecosystems to chemical pollution and GC in different biogeographic regions of Europe.

•    Develop and test new concepts for assessing the effects of complex chemical mixtures on ecosystem structure and functioning.

•    Develop a set of *in silico* tools capable of integrating GC stressors on the risk assessment of chemicals at different levels of biological organisation.

**ESR 7: Combined effects of stress posed by global climate change and chemicals**

In the context of global climate change, increased mean temperatures and a lower water pH can be expected ( Masson-Delmotte *et al.*, 2021). Besides these gradual environmental changes, there is growing empirical and computational evidence of increases in the severity and frequency of extreme weather events, such as heatwaves or warm spells, until the end of this century ( Donat & Alexander, 2012; Meehl & Tebaldi, 2004; Woolway *et al.*, 2021). Climate-chemical effect studies have mainly applied increased mean temperatures in constant temperature regimes ( Holmstrup *et al.*, 2010; Noyes *et al.*, 2009), instead of environmentally realistic daily temperature fluctuations, including seasonal variations ( Willming & Maul, 2016; Willming *et al.*, 2013). However, with these fluctuations having more adverse effects than elevated mean temperatures on species under chemical stress, the importance of temperature variability and high environmental realism is highlighted ( Vasseur *et al.*, 2014; Verheyen & Stoks, 2019). Therefore, a constant temperature regime might not be representative of exposure conditions in natural habitats where temperature variation and extreme events may occur, now and in the future.

In contrast to climate change-driven temperature fluctuations, which have been relatively intensively investigated in aquatic systems, far less is known about how changes in dissolved CO2 concentrations affect the ecology of freshwater systems ( Andersen *et al.*, 2005; Christensen *et al.*, 2006). To our knowledge, no studies have examined the combination of CO2 and chemical stressors on freshwater populations, communities, or ecosystems, although the effects of CO2 increases mediated by ocean acidification have been investigated ( Hasler *et al.*, 2018).

Research under ECORISK2050 will focus on enabling the manipulation of GC-influenced environmental variables in mesocosms using a mobile, multifunctional device. The device will be used to assess the sensitivity and recovery capacity of freshwater populations and communities exposed to pesticides and future GC-scenarios, in mesocosms along a south-to-north gradient in Europe. Finally, these experiments will be used to identify different adaptive capacities and sensitivities to GC-relevant combinations of multiple stressors.

**ESR 8: Multiple stressor effects on ecosystem vulnerability**

This research project investigates the interactions between GC and toxic chemicals in modelled and experimental freshwater systems at the population, community, and ecosystem level. The examination of the responses to multiple stress sources at different levels of biological organisation will be conducted through several micro- and mesocosm experiments simulating both lentic and lotic conditions. By doing so, peculiar features of the aquatic communities and populations, such as resistance, recovery and resilience will be studied under future exposure conditions and using additional stressors (e.g., eutrophication, hydrological stress). Eventually, the multidimensional concept of ecological stability ( Donohue *et al.,* 2013) will be used for the first time to quantify multiple stressor effects on populations and communities. This approach will be used to derive recommendations for future ecosystem management.

Alongside the experimental work, a new modelling framework will be developed. The framework will be based on the common currency of biological traits. Biological traits have recently been used to assess the sensitivity of aquatic invertebrates to both known toxic chemicals and GCC variables separately ( Sandin *et al.,* 2014; Van den Berg *et al.,* 2019). Taking advantage of the collaboration and expertise of the project partners, data and models will be combined to develop a new modelling framework, able to assess aquatic invertebrates’ vulnerability to the combined effects of climate change and chemicals by 2050 at the European level. Particularly, maps describing changes in the relative portion of invertebrates that are sensitive to chemicals with different modes of action in different geographic areas will be delivered. The framework will be composed of several steps and data will be gathered from numerous databases. First, present climatic and hydrological conditions will be studied and used as a baseline to produce predictions on future climatic and hydrological variables ( Schneider *et al.,* 2013). Then, data on present species distribution will be combined with the predicted future environmental conditions to assess how species distribution is likely to change by 2050. The derived future species distribution will be used to predict the vulnerability of aquatic invertebrates to chemicals with different modes of action by 2050, based on species traits related to sensitivity and population recovery.

**ESR 9: Effects of chemical mixtures on microbial communities**

Microbes are the largest part of the earth’s biomass and are key components of biogeochemical processes ( Falkowski *et al.,* 2008). Disturbance of microbial services can thus cause significant harm to environmental systems. Microbial communities have a high level of diversity and can rapidly modify their energetic performance and activity to respond to changing environmental conditions, including chemical pollution or GC stressors ( Acosta-González & Marqués, 2016). Given the overall good understanding of bacterial cells, their short generation times, and the ease of studying bacterial responses in the laboratory, bacteria offer an excellent, yet underutilised, approach for assessing the biological activity of chemical pollutants in a changing climate ( Girotti *et al.*, 2008; van Wezel & Opperhuizen, 1995).

Recently published studies report the effects of single contaminants on microbial communities, but knowledge about impacts of the combination of chemicals occurring in the environment is still scarce ( Milan *et al.*, 2018; Yan *et al.*, 2018). The two main reasons for this lack of knowledge are: 1) measuring concentrations of all potential contaminants in the environment is unrealistic due to the technical challenges and analytical costs, and 2) the sum of measured concentrations for different chemicals does not provide information about the exposure level of the mixture, because toxic concentrations are compound-specific. This makes the exposure, hazard, and risk assessments of chemical mixtures in the environment extremely challenging ( Gobas *et al.,* 2018). The concept of ‘chemical activity’ offers a way to overcome these constraints for chemical mixtures of neutral chemicals at concentrations below their specific toxic concentration, by enabling the conversion of concentrations of various chemicals into a common, unitless currency ( Gobas *et al.,* 2018). In such cases, when many neutral organic chemicals are present at low concentrations, additive toxicity is often observed ( Escher *et al.,* 2002). This holds true even when the substances are not related chemically or exhibit different modes of action when acting alone at acute levels ( Escher & Hermens, 2002). This phenomenon is defined as baseline toxicity, or narcosis ( Escher *et al.,* 2002) and is related to disturbances in cell membrane functioning ( van Wezel & Opperhuizen, 1995). As more hazardous, persistent, and toxic chemicals are expected to be used worldwide, the concept of chemical activity offers an integrative tool to quantify the biological potency of chemical mixtures. During this project, experiments will be implemented to analyse mixture effects on the structure and function of microbial communities to open the way for a better understanding of the effects of chemical mixtures in a changing environment.

**ESR 10: Modelling the interaction between climate change and chemical effects**

Ecosystems in the real world are subject to a variety of stressors acting on different levels of biological organisation (individual, population, community, and ecosystem). Empirically examining all possible stressor interactions at all biological levels is constrained by time and resources, even when focus is limited to freshwater systems. Thus, supplementary to the experimental studies approach, *in silico* modelling approaches are needed to detect important mechanistic links between multiple stressors (i.e., chemicals and GC) and their effects on freshwater ecosystems, and support ecological risk assessments.

Mechanistic effect models, like toxicokinetic-toxicodynamic modelling approaches, are becoming more accepted in the field of environmental risk assessment ( Ockleford *et al.,* 2018). While these approaches focus on chemical stressors, GC-related stressors are rarely included. However, exposure to chemicals at higher temperatures can alter toxicokinetics, resulting in observations of increased or reduced effects on the exposed organism ( Harwood *et al.,* 2009). Furthermore, chemical, and GC-related stressors can affect different levels of biological organisation directly or indirectly ( Bracewell *et al.,* 2019). It is therefore important to understand the joint effects caused by chemicals and GC-related stressors as well as how they can be transferred from lower to higher levels of biological organisation.

This research aims to extend existing modelling frameworks (e.g., toxicokinetic-toxicodynamic modelling and DEBtox —dynamic-energy-budget theory) to enable predictions under future multiple stressor scenarios. To that end, a modelling tool for assessing the combined effects of temperature and pesticides on individual freshwater invertebrates will be developed. In a bottom-up approach, individual responses will be propagated to higher levels of biological organisation, using individual-based models like ChimERA ( De Laender *et al.,* 2014). Additionally, theoretical modelling approaches will be used to investigate the role of intra- and inter-species relationships in population and community response to chemical and GC-related stressors. Ultimately, these tools could be implemented in chemical effect assessment by preceding and informing experimental investigations through extrapolations to future climate conditions.

***Work Package 6: Risks and mitigation*.** ERAs are essential to assess the potential consequences of exposure to hazardous chemicals for natural ecosystems, as a basis for informed risk management decisions. In Europe, ERAs need to be carried out according to chemical legislations, which are based on the precautionary principle to safeguard human and environmental health ( EFSA, 2013). An ERA is usually defined in probabilistic terms, such as "evaluating how likely it is that the environment may be impacted as a result of exposure to one or more environmental stressors [...]" ( EPA, 2014). Nevertheless, traditional ERA frameworks do not estimate the probability of adverse outcomes, but rather a single-value risk quotient: the ratio of the measured or predicted environmental concentration (PEC) of a chemical to the predicted no-effect concentration (PNEC) ( EEA, 1998). The PNEC corresponds to the highest concentration at which no adverse effects are observed in any tested species, modified by various assessments factors to account for limited information and other sources of uncertainties. This outcome has a binary interpretation: if the risk quotient for a given scenario exceeds 1, then the substance is considered to present a potential risk to the environment, otherwise the substance is considered safe.

Although such risk assessment frameworks have been used for decades, they have also been criticized for being opaque and handling uncertainties in subjective ways ( Verdonck *et al.,* 2003). Moreover, regulatory ERAs are traditionally based on single substances using single species tests performed in controlled lab conditions, and therefore must be extrapolated to assess and predict risks that chemicals and their mixtures pose to the real environment ( Topping *et al.,* 2020). Current risk assessment methods often include some probabilistic components, such as the species sensitivity distribution to characterise effects ( Posthuma *et al.,* 2019). However, past efforts to apply fully probabilistic methods for risk calculation, accounting for uncertainty in both exposure and effects, have posed challenges for calculation, visualisation and communication to stakeholders ( Jager *et al.,* 2001).

Bayesian networks (BNs), a graphical probabilistic modelling tool, are a promising method for improving ERA ( Hart & Pollino, 2008). However, despite many recent examples (reviewed by Kaikkonen *et al.,* 2021), the method is still not incorporated into regulatory frameworks. In this work package, we will explore the potential of BN modelling to explicitly account for uncertainties in both exposure and effects, from both single chemicals and mixtures, from both agricultural and urban sources. This approach will also include uncertainty in projections from future climate and demographic scenarios (WP 3).

Furthermore, integrating risk mitigation options into ERAs, i.e., a solution-focused approach has been introduced to improve the utility of ERAs and to ensure that adequate risk management decisions are taken ( Munthe *et al.,* 2019). The BN models to be developed for risk characterisation within ECORISK2050 will be extended to incorporate such alternative decisions and their effectiveness ( Carriger & Newman, 2012), and thereby function as decision-supporting tools.

The research aims of this WP are:

- Develop and apply a probabilistic model for risk assessment of agricultural chemicals under current and future GC scenarios.
- Apply probabilistic models for assessment of PPCP from wastewater emissions under current and future GC scenarios.
- Assess the effectiveness of different mitigation options to improve future wastewater quality.

**ESR 11: Probabilistic risk assessment of pesticides and their mixtures under global change scenarios**

Within the EU, ERA of pesticides is conducted on individual products using a framework of tiers of increasing specificity and decreasing conservatism. If an overestimate of environmental concentrations, for instance, produces a value far below levels at which a chemical is predicted to cause adverse effects, then typically no further assessment is needed for the substance to be authorised ( EFSA, 2013).

The traditional approach, conducted on a substance-by-substance basis using surrogate species under regulatory frameworks, is considered protective for all species. Probabilistic ERA can be used to move to a more holistic approach in ERA and overcome some of the shortcomings of today’s prospective and retrospective frameworks, as they lack consideration of real spatial and temporal exposure, and widely exclude the assessment of multiple chemical (mixture) exposure as well as other factors that influence the exposure to or effects of these chemicals ( Topping *et al.,* 2020).

While current probabilistic approaches use the probability distribution to extract a single number to enter into the risk quotient ( Posthuma *et al.,* 2019), less attention is paid to risk uncertainty intervals or the visualisation of risk ( Verdonck *et al.,* 2003). This project will use BNs that can overcome these limitations by simultaneously integrating multiple assessment endpoints, multiple stressors, and permitting the better consideration of mixtures’ risks to the environment. BNs offer further flexibility in data sources ( Chen & Pollino, 2012), incorporating direct measurements or output from models ( Pitchforth & Mengersen, 2013) even where empirical data are absent, using expert opinions to establish baseline information and establish the probability and magnitude of environmental and anthropogenic effects ( Stelzenmüller *et al.,* 2018). The flexibility of BNs means that they can be a useful tool to assess the risks of pesticides to the environment, by incorporating probability distributions in the model to derive risk estimate distributions. They can also be developed towards risk assessment of pesticide mixtures, by incorporating mathematical methods currently in use and distributions for both the effect and exposure parts. Additionally, the BN model can be used to combine the output of pesticide prediction models (e.g., WISPE) that account for future climate projections, crops scenarios, and pesticide exposure models, with risk estimation calculations that predict ecological risk of pesticides under future conditions, while also integrating the uncertainties quantified as probability distributions at each step.

**ESR 12: Probabilistic risk assessment of pharmaceuticals in the freshwater environment**

The regulation of pharmaceuticals in Europe has long been driven by medical needs, leaving environmental risk assessment a largely descriptive measure, based on the comparison of lab-extrapolated effect thresholds and concentrations predicted from market penetration and dose sizes ( EMA, 2018). As demographic and climatic shifts drive intensifying and diversifying stressors in the aquatic environment, the importance of effective and comprehensive risk assessment approaches that adequately account for future uncertainty can only grow.

Predicting environmental concentrations from pharmaceutical sales data remains a potential alternate approach, where data are available ( Grung *et al.,* 2008). In Norway, the Norwegian Institute of Public Health collects spatially and temporally categorised data, allowing predicted concentrations to be informed by real-world variation in use. By comparing conventional predicted concentrations to sales-based predictions and measured concentrations, we can better quantify uncertainty in the emission-to-toxicity pathway. Furthermore, through explicit modelling of these pathways, we can also consider uncertainty in persistence of pollutants as part of the overall risk profile, rather than employing a simple numerical cut-off point.

In the ESR 12 project, we will develop a probabilistic model integrating these pathways, their uncertainty, and the overall risk assessment process within a Norwegian setting, following the Bayesian network risk assessment approach described for ESR 11. It is then planned that a more integrated risk assessment framework can be developed ( Sperotto *et al.,* 2017), allowing for better modelling and communication of the relationships between urbanisation and demographic changes, pharmaceutical pollution, and environmental changes. This approach can be used to deliver a risk-based prioritisation list of pharmaceutical pollutants in Norway as well as in other European countries.

**ESR 13: Mitigation options to reach a toxic-free environment**

It is evident that effective mitigation options are needed to reduce chemical emissions into the environment. Currently, the main focus is on wastewater treatment to remove chemicals. However, considering the limits of technological treatments, other options are urgently needed ( Kümmerer *et al.,* 2018; van Wezel *et al.,* 2017). Within ECORISK2050, options covering the design, authorisation, use and waste stage of chemicals will be assessed.

During the chemical design, the concept of ‘green chemistry’ can be applied to develop less hazardous chemicals with increased biodegradability, whilst their initial function is maintained. Recently, Keijer *et al.* (2019) proposed a framework for ‘circular chemistry’, by expanding the principles of green chemistry to the entire life cycle of chemical products and considering by-products as a resource instead of waste. Implemented correctly, circular chemistry would not only minimise chemical risks but could also support the European strategy for a circular economy, among others.

Authorisation of chemicals in the EU is managed through different legislative frameworks which are organised per market type ( van Dijk *et al.,* 2021). Within these frameworks, an ERA for the aquatic environment needs to be submitted as part of the chemical registration requirements. Risk assessment outcomes are used to manage substances prior to placement on the market through classification, labelling, restrictions, or outright bans on usage of chemicals. Current risk assessments need to be adjusted as assessments are ill-equipped to deal with important environmental and economic phenomena including mixture toxicity, other chemical uses than those assessed, and regrettable substitution, where banned substances are replaced with new, potentially more harmful chemicals. Furthermore, as a given substance can be regulated under multiple legislative frameworks and environmental risk assessment procedures between frameworks differ, there is potential for inconsistency among marketing authorisation for chemicals.

Chemicals are used both professionally — for instance, pesticides in agricultural practices, pharmaceuticals in hospitals — and non-professionally, like biocides in household products. Mandatory mitigation options are only available for professional users. Environmental risks of chemicals are currently underestimated due to unregulated emissions from non-professional use ( Wieck *et al.,* 2018), highlighting the importance of the sustainable use of chemicals by non-professional users, too. Reduced and sustainable use of chemicals can be achieved by applying the principles of ‘essential use’ to phase out certain applications ( Cousins *et al.,* 2019).

Lastly, technological treatment options of wastewater will be assessed, as these will remain indispensable since chemical emissions to the environment cannot always be prevented. The ECORISK2050 project will seek to determine an appropriate combination of mitigation options across the whole chemical life cycle, to close the gap to a toxic-free environment.

## Dissemination and outreach

The ECORISK2050 project is actively developing contacts with the wider public and scientific community. We aim to:

- Disseminate results via scientific publications and conferences, and through stakeholder and end-user-oriented workshops.
- Produce policy briefs for government agencies, regional authorities and other public bodies involved in policy-making such as the European Commission, ECHA, EFSA, EMA, JRC, EEA and OECD, as well as for National authorities in the countries of the participating organisations.
- Produce advice notes for companies outlining the results produced and the key issues that companies should consider.
- Provide information to the public on the ECORISK2050 website and through articles and features for popular science magazines in each country.
- Provide information to school pupils through open days and ESR participation in the school liaison activities of their host institutions.

We are also producing a series of Massive Open Online Courses (MOOCs) which are placed on the website, while the twitter account @EcoITN is used as the main channel to publish (weekly) updates about ongoing and finished work of the ESRs, conferences and group meetings. ECORISK2050 is coordinated by Paul van den Brink ( paul.vandenbrink@wur.nl).

## Abbreviations

ESR = early stage researcher, GC = global change, IPCC = Intergovernmental Panel on Climate Change, RCP = representative concentration pathway, SSP = shared socio-economic pathway, PPCP = pharmaceuticals and personal care products, WP = work package, TKTD = toxicokinetic-toxicodynamic, GCC = global climate change, ERA = environmental risk assessment

## Data availability

No data are associated with this article.
