# Supplementary material for: ECORISK2050: An Innovative Training Network for predicting the effects of global change on the emission, fate, effects, and risks of chemicals in aquatic ecosystems
Source: Open Res Eur. 2022 May 16;1:154. Originally published 2021 Dec 20. [Version 2] doi: 10.12688/openreseurope.14283.2 (PMC10446038; doi:10.12688/openreseurope.14283.2)
Supplement: Peer review history

# Peer review: Reviewer response for version 2

**DOI:** 10.21956/openreseurope.16002.r29285

**Published:** 2022-06-07

**Authors:** Hans-Christian Bruun Hansen

**Affiliations:** 1 Department of Plant and Environmental Sciences, University of Copenhagen, Frederiksberg, Denmark

I think it is fine. It can be somewhat difficult for the reader to very clearly pinpoint what is the exactly new thing about the project compared with previous risk assessments. Personally, I think that the development is going faster than here anticipated and the chemicals we will deal with in the future probably may not be classical pesticides, pharmaceuticals and similar. We are seeing a dramatic food shift, the green transition will change the industry towards biological products, new soil amendments will appear, and so on. It could be interesting to have a stronger delineation of that and what are the chemicals that the future will deal with. So a last thing could be to revise the abstract so i) the novelty of the project is clearer, and ii) what are the novel chemicals and scenarios not known today that will be dealt with.

Where applicable, are recommendations and next steps explained clearly for others to follow? (Please consider whether others in the research community would be able to implement guidelines or recommendations and/or constructively engage in the debate)

Partly

Does the article adequately reference differing views and opinions?

Yes

Are all factual statements correct, and are statements and arguments made adequately supported by citations?

Yes

Is the rationale for the Open Letter provided in sufficient detail? (Please consider whether existing challenges in the field are outlined clearly and whether the purpose of the letter is explained)

Yes

Is the Open Letter written in accessible language? (Please consider whether all subject-specific terms, concepts and abbreviations are explained)

Yes

Reviewer Expertise:

NA

I confirm that I have read this submission and believe that I have an appropriate level of expertise to confirm that it is of an acceptable scientific standard.

# Peer review: Reviewer response for version 1

**DOI:** 10.21956/openreseurope.15410.r28241

**Published:** 2022-02-18

**Authors:** Hans-Christian Bruun Hansen

**Affiliations:** 1 Department of Plant and Environmental Sciences, University of Copenhagen, Frederiksberg, Denmark

The paper describes the idea and objectives of an EU ITN training network called Ecorisk2050 with a focus on how global change affects the emission, fate, effects and risks of chemicals in aquatic ecosystems.

 The paper provides a short background and formulation of the main objectives and anticipated results, and the project structure, and then runs through the work that each ESR has to do. A lot of focus on models and scenarios.

 It is difficult to criticize or comment on the content - as it is. However, it is very generic and as a reader, I got lost in all the text and the lack of concrete content, directions or work. The work as it is outlined seems to require not just 13 ESRs, but hundreds of people - it is overwhelming. As I guess the project has been running since late 2019 or early 2020 there must be some concrete lines of thinking and results. And more graphics could be produced to help understand what are the novel parts. It should be possible to show figures or graphics where you have global stressors and then the major impacts you are investigating.

 I couldn't clearly understand how climate effects on cropping patterns and crop migration in Europe affects agrochemicals dispersion and fate. The green transition agenda is moving fast - so where are all the "biologicals" and gene technologies used in biopesticide approaches? Cropping patterns are shifting fast as farmers have to use more leguminous protein-producing crops - this may have many consequences.

 You may also help the reader on the model side. If you are not experienced with models, then you get lost here. So what are the conceptual approaches and the novelties that you introduce in order to be able to handle and model the global changes? Most of the agricultural contaminants have passed soil systems before they are seen in rivers, lakes or groundwater. Where are fate processes in soils as affected by climate (which ESRs) - must be one of the most critical points.

 For urban environments flooding and flushes must be critical, but how is it included?

 Another development that is running fast is the analytical capabilities - non-target approaches I guess will be the standard in 10 years. So talking about 2050 -  how do we get the new analytical capabilities and data explosion included in risk assessment? So regulation may change substantially as the list of risk chemicals may grow exponentially.

 The dissemination activities, the training activities and the collaboration between academia and industry needs more emphasis. When reading this paper how can the reader get insight, learn, get involved and be trained? And how will regulation and public authorities as well as other stakeholders get on board?

**In conclusion**:

 An interesting topic and ITN - very relevant and set for a lot of interesting research. However, for the reader, it is difficult to get much concrete from the paper as it is too generic. I suggest you try to work with figures and graphics to communicate your ideas, and to include examples/boxed to make things concrete.

Where applicable, are recommendations and next steps explained clearly for others to follow? (Please consider whether others in the research community would be able to implement guidelines or recommendations and/or constructively engage in the debate)

Partly

Does the article adequately reference differing views and opinions?

Yes

Are all factual statements correct, and are statements and arguments made adequately supported by citations?

Yes

Is the rationale for the Open Letter provided in sufficient detail? (Please consider whether existing challenges in the field are outlined clearly and whether the purpose of the letter is explained)

Yes

Is the Open Letter written in accessible language? (Please consider whether all subject-specific terms, concepts and abbreviations are explained)

Yes

Reviewer Expertise:

Environmental Science and Technology, Chemosphere, Chem. Engin. J., Water Research, J. Hazardous Materials, Environtal Toxicology and Chemistry, J. Agricultural and Food Chemisry, and many others

I confirm that I have read this submission and believe that I have an appropriate level of expertise to confirm that it is of an acceptable scientific standard, however I have significant reservations, as outlined above.

# Response

**Published:** 2022-05-09

**Authors:** Sam Welch

**Affiliations:** Norwegian institute for water research, Norway

Thank you for your review of our paper. We've attempted to address the following feedback:

***As I guess the project has been running since late 2019 or early 2020 there must be some concrete lines of thinking and results. And more graphics could be produced to help understand what are the novel parts. It should be possible to show figures or graphics where you have global stressors and then the major impacts you are investigating.***

 Deliverables and outputs, including novel published work so far, are summarised later in the paper. Two additional figures have been added, summarising a) the overall project and ESRs and b) probabilistic risk assessment.

***I couldn't clearly understand how climate effects on cropping patterns and crop migration in Europe affects agrochemicals dispersion and fate. The green transition agenda is moving fast - so where are all the "biologicals" and gene technologies used in biopesticide approaches? Cropping patterns are shifting fast as farmers have to use more leguminous protein-producing crops - this may have many consequences.***

 ESR11 addresses in the last paragraph interactions between climate effects and agrochemical dispersion and fate; more information can be found in their papers. Biopesticide approaches are largely outside of the scope of the project, as a work addressing chemical pollution.

***You may also help the reader on the model side. If you are not experienced with models, then you get lost here. So what are the conceptual approaches and the novelties that you introduce in order to be able to handle and model the global changes? Most of the agricultural contaminants have passed soil systems before they are seen in rivers, lakes or groundwater. Where are fate processes in soils as affected by climate (which ESRs) - must be one of the most critical points.***

 Planned modelling of global changes largely involves the employment of existing scenarios and approaches to answer the questions posed in the paper, rather than novel approaches. ESR3 hopes to include some consideration of climate-driven effects on fate processes in soils, while ESR 11 is currently working to combine Bayesian Networks and the WISPE exposure prediction model, which includes climate conditions and their effect on the pesticide exposure via fate and transport in soil, although this is not ready yet for publication.

***For urban environments flooding and flushes must be critical, but how is it included?***

 We agree with reviewer comment that flooding and flushes are critical for urban environments, especially for specific environments like York where these problems already occur. Future climatic events including flooding, rainfall or droughts are included in the RCPs scenarios. In the developments of our scenarios, RCPs climate events data will be included and therefore considered on the future chemical emissions in urban environments. Phrasing of the manuscript has been updated to clarify this.

***Another development that is running fast is the analytical capabilities - non-target approaches I guess will be the standard in 10 years. So talking about 2050 - how do we get the new analytical capabilities and data explosion included in risk assessment? So regulation may change substantially as the list of risk chemicals may grow exponentially.***

 Although this is an important issue, ESR 11 and 12's work focuses on mainly on prospective risk assessment, using past data to predict future risk. Probabilistic risk assessment may be better placed to address more complex data, but ultimately the scope of the ECORISK 2050 project does not include these particular future concerns.

***The dissemination activities, the training activities and the collaboration between academia and industry needs more emphasis. When reading this paper how can the reader get insight, learn, get involved and be trained? And how will regulation and public authorities as well as other stakeholders get on board?***

 We've sought to address this request by adding tables summarising: a) government, industry and academic partners and b) online courses delivered as part of the project's outreach work. Although the training element of this project has largely completed, we have updated the Results and Dissemination section to clarify our remaining planned work, hosting a concluding workshop to present to stakeholders in October 2022 and the creation of policy briefs.

# Peer review: Reviewer response for version 1

**DOI:** 10.21956/openreseurope.15410.r28239

**Published:** 2022-01-26

**Authors:** Luděk Bláha

**Affiliations:** 1 Faculty of Science, RECETOX, Masaryk University, Brno, Czech Republic

This Open letter introduces and summarizes the objectives and (planned) activities of the ECORISK2050 ITN. This is an ambitious project with several components and complex outcomes. The text is well elaborated, it is informative, and provides a number of inspirations for readers. In addition to outlining the challenges, it also provides reasonable approaches how to tackle them (namely through individual projects of ESRs).

 The following points could be considered:

 The text ends with the Dissemination and Outreach section (and there are no Conclusions). I think that reader would benefit from having a brief or illustrative conclusions/summary at the end. It could, for example, provide some highlights (e.g. highlight limited number of the most important or practical outcomes/tools, etc).

 The ITN runs since 2019, and there are certainly already some solid results/outcomes. The text is written in the future time (what will be done) but it might be beneficial to provide a current update on what has been actually achieved so far.

Where applicable, are recommendations and next steps explained clearly for others to follow? (Please consider whether others in the research community would be able to implement guidelines or recommendations and/or constructively engage in the debate)

Yes

Does the article adequately reference differing views and opinions?

Yes

Are all factual statements correct, and are statements and arguments made adequately supported by citations?

Yes

Is the rationale for the Open Letter provided in sufficient detail? (Please consider whether existing challenges in the field are outlined clearly and whether the purpose of the letter is explained)

Yes

Is the Open Letter written in accessible language? (Please consider whether all subject-specific terms, concepts and abbreviations are explained)

Yes

Reviewer Expertise:

Environmental Toxicology

I confirm that I have read this submission and believe that I have an appropriate level of expertise to confirm that it is of an acceptable scientific standard.

# Response

**Published:** 2022-05-06

**Authors:** Sam Welch

**Affiliations:** Norwegian institute for water research, Norway

Thank you for your review of our paper. We've addressed the following feedback:

***The text ends with the Dissemination and Outreach section (and there are no Conclusions). I think that reader would benefit from having a brief or illustrative conclusions/summary at the end. It could, for example, provide some highlights (e.g. highlight limited number of the most important or practical outcomes/tools, etc).***

***The ITN runs since 2019, and there are certainly already some solid results/outcomes. The text is written in the future time (what will be done) but it might be beneficial to provide a current update on what has been actually achieved so far.***

 Tables have been added, summarising the journal articles and project deliverables published at time of writing, as well as the now concluded series of Massive Open Online Courses organised by the project and now freely available online.
